# 5,5′-Bis(naphthalen-2-yl)-2,2′-bi(1,3,4-oxadiazole)

**DOI:** 10.1107/S1600536811048513

**Published:** 2011-11-19

**Authors:** Haitao Wang, Xiaoshi Jia, Songnan Qu, Binglian Bai, Min Li

**Affiliations:** a Key Laboratory of Automobile Materials (MOE), College of Materials Science and Engineering, Jilin University, Changchun 130012, People’s Republic of China; b Key Laboratory of Excited State Processes, Changchun Institute of Optics, Fine Mechanics and Physics, Chinese Academy of Sciences, Changchun 130033, People’s Republic of China; c College of Physics, Jilin University, Changchun 130012, People’s Republic of China

## Abstract

The title mol­ecule, C_24_H_14_N_4_O_2_, lies on an inversion centre and the asymmetric unit containg one half-mol­ecule. The naphthalene ring systems are twisted slightly with respect to the oxadiazole rings, making a dihedral angle of 1.36 (6)°. These mol­ecules are π-stacked along the crystallographic *a* axis, with an inter­planar distance of 3.337 (1) Å. Adjacent mol­ecules are slipped from the ‘ideal’ cofacial π-stack in both the long and short mol­ecular axis (the long mol­ecular axis is defined as the line through the naphthalene C atom in the 6-position and the mol­ecular center, the short mol­ecular axis is in the mol­ecular plane perpendicular to it). The slip distance along the long mol­ecular axis (*S*_1_) is 7.064 (1) Å, nearly a two-ring-length displacement. The side slip (*S*_2_, along the short mol­ecular axis) is 1.159 (8) Å.

## Related literature

For the synthesis of 1,3,4-oxadiazole derivatives: see Schulz *et al.* (1997 ▶). For related structures: see Schulz *et al.* (2005 ▶); Qu *et al.* (2008 ▶); Landis *et al.* (2008 ▶).
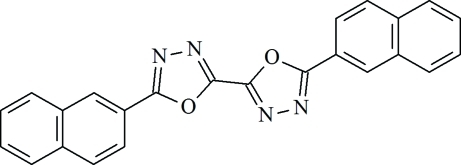


## Experimental

### 

#### Crystal data

C_24_H_14_N_4_O_2_
*M* *_r_* = 390.39
Monoclinic, 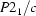

*a* = 7.8982 (16) Å
*b* = 5.7107 (11) Å
*c* = 21.503 (5) Å
β = 109.82 (3)°
*V* = 912.4 (3) Å^3^
*Z* = 2
Mo *K*α radiation
μ = 0.09 mm^−1^
*T* = 293 K
0.18 × 0.14 × 0.12 mm

#### Data collection

Rigaku R-AXIS RAPID diffractometer
Absorption correction: multi-scan (*ABSCOR*; Higashi, 1995 ▶) *T*_min_ = 0.983, *T*_max_ = 0.989
8518 measured reflections
2091 independent reflections
1468 reflections with *I* > 2σ(*I*)
*R*_int_ = 0.030

#### Refinement

*R*[*F* ^2^ > 2σ(*F* ^2^)] = 0.039
*wR*(*F* ^2^) = 0.106
*S* = 1.07
2091 reflections
136 parameters
H-atom parameters constrained
Δρ_max_ = 0.16 e Å^−3^
Δρ_min_ = −0.18 e Å^−3^

### 

Data collection: *RAPID-AUTO* (Rigaku, 1998 ▶); cell refinement: *RAPID-AUTO*; data reduction: *RAPID-AUTO*; program(s) used to solve structure: *SHELXS97* (Sheldrick, 2008 ▶); program(s) used to refine structure: *SHELXL97* (Sheldrick, 2008 ▶); molecular graphics: *PLATON* (Spek, 2009 ▶) and *CrystalStructure* (Rigaku/MSC, 2002 ▶); software used to prepare material for publication: *SHELXL97*.

## Supplementary Material

Crystal structure: contains datablock(s) I, global. DOI: 10.1107/S1600536811048513/vm2137sup1.cif

Structure factors: contains datablock(s) I. DOI: 10.1107/S1600536811048513/vm2137Isup2.hkl

Additional supplementary materials:  crystallographic information; 3D view; checkCIF report

## Figures and Tables

**Fig. 1.**
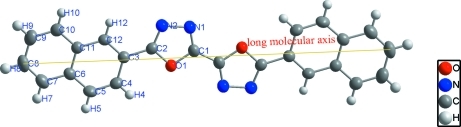
The molecular structure of the title compound with displacement ellipoids drawn at the 50% probability level. The asymmetric unit only contains a half molecule, the second half is generated by symmetry code -x, -y+1, -z+2. The line through C8 and the molecular center is defined as the long molecular axis.

**Fig. 2.**
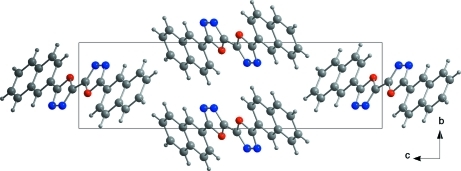
Molecular packing as viewed down the crystallographic a axis.

**Fig. 3.**
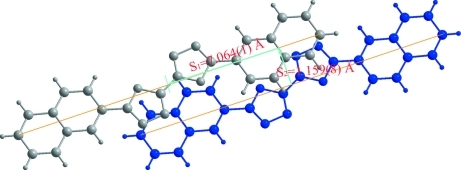
Two adjacent molecules in the molecular stacks as viewed perpendicular to the molecular plane. The slip distances along the long molecular axis (S1) and short axis (S2) are 7.064 (1)Å and 1.159 (8) Å, respectively.

## supplementary crystallographic information

### Comment

Aromatic heterocycles, such as 1,3,4-oxadiazole and thiophene rings, which are conjugatable to phenyl rings, are often directly connected to the phenyl ring to obtain a large *π*-conjugated system or to tune the electronic structure. These compounds are of interest as charge transport materials or emitting layers in electroluminescent diodes (Schulz *et al.*, 1997, Schulz *et al.*, 2005). Comparing to thiophene derivatives, 1,3,4-oxadiazole derivatives are more likely to form *π*-stacked molecular packing (Schulz *et al.*, 2005, Qu *et al.*, 2008, Landis *et al.*, 2008).

As shown in Fig. 1, both 1,3,4-oxadiazole rings are in a *trans*-conformation, which yields a linear molecular shape. These molecules are *π*-stacked along the crystallographic *a*-axis (Fig. 2). The molecules in the stacks are canted relative to the stacking axis by 26.57 (1)°. Adjacent molecules are slipped off each other in both long and short molecular axis to avoid unfavorable electrostatic interactions in the "ideal" cofacial stacks (Fig. 3).

### Experimental

The tile compound was synthesized through a two-step reaction. Firstly, naphthylacyl hydrazide was reacted with oxalyl chloride in THF at room temperature for 8 h, yielding the product, oxalyl acid *N',N'*-di-naphthylacyl hydrazide. Secondly, the title compound was derived by intramolecular cyclization of this dihydrazide derivative with POCl_3_ under reflux conditions, and the coarse product was further purified by washing with DMSO for the 1H NMR FT—IR spectroscopic characterization and elemental analysis. Yield >70%. Crystals of the title compound suitable for X-ray diffraction were obtained by a slow diffusion method (diethyl ether was diffused into chloroform solution).

### Refinement

Carbon-bound H-atoms were placed in calculated positions with C—H = 0.93 Å and were included in the refinement in the riding model with *U*_iso_(H) = 1.2 *U*_eq_(C).

### Crystal data

**Table:**

C_24_H_14_N_4_O_2_	*Z* = 2
*M**_r_* = 390.39	*F*(000) = 404
Monoclinic, *P*2_1_/*c*	*D*_x_ = 1.421 Mg m^−^^3^
Hall symbol: -P 2ybc	Mo *K*α radiation, λ = 0.71073 Å
*a* = 7.8982 (16) Å	µ = 0.09 mm^−^^1^
*b* = 5.7107 (11) Å	*T* = 293 K
*c* = 21.503 (5) Å	Block, colourless
β = 109.82 (3)°	0.18 × 0.14 × 0.12 mm
*V* = 912.4 (3) Å^3^	

### Data collection

**Table:**

Rigaku R-AXIS RAPID diffractometer	2091 independent reflections
Radiation source: fine-focus sealed tube	1468 reflections with *I* > 2σ(*I*)
graphite	*R*_int_ = 0.030
ω scans	θ_max_ = 27.5°, θ_min_ = 3.7°
Absorption correction: multi-scan (*ABSCOR*; Higashi, 1995)	*h* = −10→10
*T*_min_ = 0.983, *T*_max_ = 0.989	*k* = −7→7
8518 measured reflections	*l* = −27→27

### Refinement

**Table:**

Refinement on *F*^2^	Primary atom site location: structure-invariant direct methods
Least-squares matrix: full	Secondary atom site location: difference Fourier map
*R*[*F*^2^ > 2σ(*F*^2^)] = 0.039	Hydrogen site location: inferred from neighbouring sites
*wR*(*F*^2^) = 0.106	H-atom parameters constrained
*S* = 1.07	*w* = 1/[σ^2^(*F*_o_^2^) + (0.0577*P*)^2^] where *P* = (*F*_o_^2^ + 2*F*_c_^2^)/3
2091 reflections	(Δ/σ)_max_ = 0.001
136 parameters	Δρ_max_ = 0.16 e Å^−^^3^
0 restraints	Δρ_min_ = −0.18 e Å^−^^3^

### Special details

**Table:**

Geometry. All e.s.d.'s (except the e.s.d. in the dihedral angle between two l.s. planes) are estimated using the full covariance matrix. The cell e.s.d.'s are taken into account individually in the estimation of e.s.d.'s in distances, angles and torsion angles; correlations between e.s.d.'s in cell parameters are only used when they are defined by crystal symmetry. An approximate (isotropic) treatment of cell e.s.d.'s is used for estimating e.s.d.'s involving l.s. planes.
Refinement. Refinement of *F*^2^ against ALL reflections. The weighted *R*-factor *wR* and goodness of fit *S* are based on *F*^2^, conventional *R*-factors *R* are based on *F*, with *F* set to zero for negative *F*^2^. The threshold expression of *F*^2^ > σ(*F*^2^) is used only for calculating *R*-factors(gt) *etc*. and is not relevant to the choice of reflections for refinement. *R*-factors based on *F*^2^ are statistically about twice as large as those based on *F*, and *R*- factors based on ALL data will be even larger.

### Fractional atomic coordinates and isotropic or equivalent isotropic displacement parameters (Å^2^)

**Table:**

	*x*	*y*	*z*	*U*_iso_*/*U*_eq_	
O1	0.18040 (11)	0.40921 (16)	0.97102 (4)	0.0412 (2)	
N1	0.01904 (14)	0.7350 (2)	0.94532 (6)	0.0482 (3)	
N2	0.14392 (14)	0.7347 (2)	0.91193 (6)	0.0479 (3)	
C1	0.04644 (15)	0.5425 (2)	0.97855 (6)	0.0407 (3)	
C2	0.23461 (16)	0.5413 (2)	0.92839 (6)	0.0388 (3)	
C3	0.38061 (15)	0.4564 (2)	0.90721 (6)	0.0373 (3)	
C4	0.46713 (17)	0.2407 (2)	0.93102 (6)	0.0448 (3)	
H4	0.4287	0.1499	0.9596	0.054*	
C5	0.60641 (17)	0.1660 (2)	0.91221 (7)	0.0453 (3)	
H5	0.6637	0.0256	0.9288	0.054*	
C6	0.66520 (15)	0.2993 (2)	0.86777 (6)	0.0389 (3)	
C7	0.80712 (17)	0.2254 (3)	0.84590 (7)	0.0506 (4)	
H7	0.8681	0.0868	0.8621	0.061*	
C8	0.85466 (18)	0.3556 (3)	0.80150 (8)	0.0586 (4)	
H8	0.9466	0.3037	0.7870	0.070*	
C9	0.76681 (19)	0.5674 (3)	0.77722 (8)	0.0565 (4)	
H9	0.8008	0.6543	0.7468	0.068*	
C10	0.63230 (17)	0.6463 (3)	0.79789 (6)	0.0459 (3)	
H10	0.5763	0.7881	0.7821	0.055*	
C11	0.57699 (15)	0.5144 (2)	0.84322 (6)	0.0364 (3)	
C12	0.43475 (15)	0.5888 (2)	0.86414 (6)	0.0379 (3)	
H12	0.3768	0.7297	0.8485	0.045*	

### Atomic displacement parameters (Å^2^)

**Table:**

	*U*^11^	*U*^22^	*U*^33^	*U*^12^	*U*^13^	*U*^23^
O1	0.0390 (4)	0.0454 (6)	0.0423 (5)	0.0017 (4)	0.0180 (4)	0.0038 (4)
N1	0.0447 (6)	0.0538 (7)	0.0506 (6)	0.0060 (5)	0.0222 (5)	0.0051 (6)
N2	0.0464 (6)	0.0498 (7)	0.0518 (6)	0.0059 (5)	0.0225 (5)	0.0066 (5)
C1	0.0343 (6)	0.0481 (8)	0.0402 (6)	0.0009 (5)	0.0133 (5)	−0.0021 (6)
C2	0.0392 (6)	0.0417 (7)	0.0358 (6)	−0.0030 (5)	0.0131 (5)	0.0017 (5)
C3	0.0363 (6)	0.0379 (7)	0.0371 (6)	−0.0013 (5)	0.0115 (5)	−0.0009 (5)
C4	0.0512 (7)	0.0399 (7)	0.0453 (7)	0.0005 (6)	0.0190 (6)	0.0083 (6)
C5	0.0481 (7)	0.0357 (7)	0.0495 (7)	0.0050 (5)	0.0130 (6)	0.0036 (6)
C6	0.0366 (6)	0.0377 (7)	0.0403 (6)	0.0001 (5)	0.0103 (5)	−0.0054 (5)
C7	0.0433 (7)	0.0485 (9)	0.0588 (8)	0.0042 (6)	0.0156 (6)	−0.0084 (7)
C8	0.0438 (7)	0.0718 (11)	0.0680 (9)	−0.0010 (7)	0.0290 (7)	−0.0128 (8)
C9	0.0522 (8)	0.0680 (11)	0.0569 (8)	−0.0083 (7)	0.0285 (7)	0.0018 (8)
C10	0.0450 (7)	0.0465 (8)	0.0472 (7)	−0.0033 (6)	0.0168 (6)	0.0036 (6)
C11	0.0363 (6)	0.0366 (7)	0.0355 (6)	−0.0036 (5)	0.0110 (5)	−0.0029 (5)
C12	0.0395 (6)	0.0340 (7)	0.0391 (6)	0.0019 (5)	0.0118 (5)	0.0026 (5)

### Geometric parameters (Å, °)

**Table:**

O1—C1	1.3568 (15)	C6—C7	1.4190 (17)
O1—C2	1.3636 (15)	C6—C11	1.4228 (18)
N1—C1	1.2889 (18)	C7—C8	1.360 (2)
N1—N2	1.4031 (16)	C7—H7	0.9300
N2—C2	1.2986 (17)	C8—C9	1.404 (2)
C1—C1^i^	1.443 (3)	C8—H8	0.9300
C2—C3	1.4588 (17)	C9—C10	1.3600 (19)
C3—C12	1.3714 (17)	C9—H9	0.9300
C3—C4	1.4175 (18)	C10—C11	1.4130 (18)
C4—C5	1.3629 (19)	C10—H10	0.9300
C4—H4	0.9300	C11—C12	1.4098 (17)
C5—C6	1.4173 (19)	C12—H12	0.9300
C5—H5	0.9300		
			
C1—O1—C2	101.84 (10)	C7—C6—C11	118.45 (12)
C1—N1—N2	105.54 (11)	C8—C7—C6	120.48 (14)
C2—N2—N1	106.25 (11)	C8—C7—H7	119.8
N1—C1—O1	113.78 (11)	C6—C7—H7	119.8
N1—C1—C1^i^	127.93 (15)	C7—C8—C9	120.87 (14)
O1—C1—C1^i^	118.28 (15)	C7—C8—H8	119.6
N2—C2—O1	112.58 (11)	C9—C8—H8	119.6
N2—C2—C3	128.24 (12)	C10—C9—C8	120.39 (14)
O1—C2—C3	119.18 (12)	C10—C9—H9	119.8
C12—C3—C4	120.03 (11)	C8—C9—H9	119.8
C12—C3—C2	119.22 (12)	C9—C10—C11	120.50 (14)
C4—C3—C2	120.75 (12)	C9—C10—H10	119.8
C5—C4—C3	120.24 (12)	C11—C10—H10	119.8
C5—C4—H4	119.9	C12—C11—C10	121.71 (12)
C3—C4—H4	119.9	C12—C11—C6	119.00 (11)
C4—C5—C6	120.93 (13)	C10—C11—C6	119.29 (11)
C4—C5—H5	119.5	C3—C12—C11	120.93 (12)
C6—C5—H5	119.5	C3—C12—H12	119.5
C5—C6—C7	122.68 (13)	C11—C12—H12	119.5
C5—C6—C11	118.86 (11)		
			
C1—N1—N2—C2	0.01 (14)	C4—C5—C6—C11	0.41 (19)
N2—N1—C1—O1	0.15 (15)	C5—C6—C7—C8	177.78 (13)
N2—N1—C1—C1^i^	179.92 (16)	C11—C6—C7—C8	−1.28 (19)
C2—O1—C1—N1	−0.23 (14)	C6—C7—C8—C9	1.1 (2)
C2—O1—C1—C1^i^	179.97 (14)	C7—C8—C9—C10	0.1 (2)
N1—N2—C2—O1	−0.16 (14)	C8—C9—C10—C11	−1.1 (2)
N1—N2—C2—C3	−179.98 (12)	C9—C10—C11—C12	−178.28 (12)
C1—O1—C2—N2	0.24 (13)	C9—C10—C11—C6	0.84 (19)
C1—O1—C2—C3	−179.92 (11)	C5—C6—C11—C12	0.36 (17)
N2—C2—C3—C12	−0.4 (2)	C7—C6—C11—C12	179.46 (11)
O1—C2—C3—C12	179.76 (10)	C5—C6—C11—C10	−178.78 (11)
N2—C2—C3—C4	179.09 (12)	C7—C6—C11—C10	0.33 (17)
O1—C2—C3—C4	−0.72 (18)	C4—C3—C12—C11	−0.42 (18)
C12—C3—C4—C5	1.19 (19)	C2—C3—C12—C11	179.11 (11)
C2—C3—C4—C5	−178.33 (12)	C10—C11—C12—C3	178.77 (11)
C3—C4—C5—C6	−1.2 (2)	C6—C11—C12—C3	−0.35 (18)
C4—C5—C6—C7	−178.66 (12)		

Symmetry codes: (i) −*x*, −*y*+1, −*z*+2.
